# Solid-State-Trapped Reactive Ammonium Carbamate Self-Derivative Salts of Prolinamide

**DOI:** 10.1002/open.201300025

**Published:** 2013-08-12

**Authors:** Anaëlle Tilborg, Steve Lanners, Bernadette Norberg, Johan Wouters

**Affiliations:** [a] Chemistry Department, University of NamurRue de Bruxelles 61, 5000 Namur (Belgium) E-mail: johan.wouters@unamur.be

**Keywords:** cocrystals, crystal structure determination, prolinamides, reactive species, ammonium carbamate self-derivative salts

## Abstract

Single crystals for two polymorphs of the ammonium carbamate self-derivative salt of prolinamide have been successfully obtained and characterized. Decarbonation of the carbamate salts was monitored by calorimetry, confirming stabilization of the reactive carbonated adducts in the solid state. Sublimation of the salts afforded crystals of prolinamide, leading to the first crystal structure of this otherwise common molecule. Reactivity of the ammonium carbamate self-derivative salt is further illustrated by the observation of a series of derived products, including dehydroprolinamide, a methylene-bridged prolinamide, and a bicyclic derivative. Crystal structures of these products display distinct amidic and/or non-amidic hydrogen bonding. This study emphasizes the reactivity of carbonated amines stabilized in the solid and opens perspectives for a systematic study of (solid-state) reactions involving these trapped reactive species.

## Introduction

**Scheme 1:**
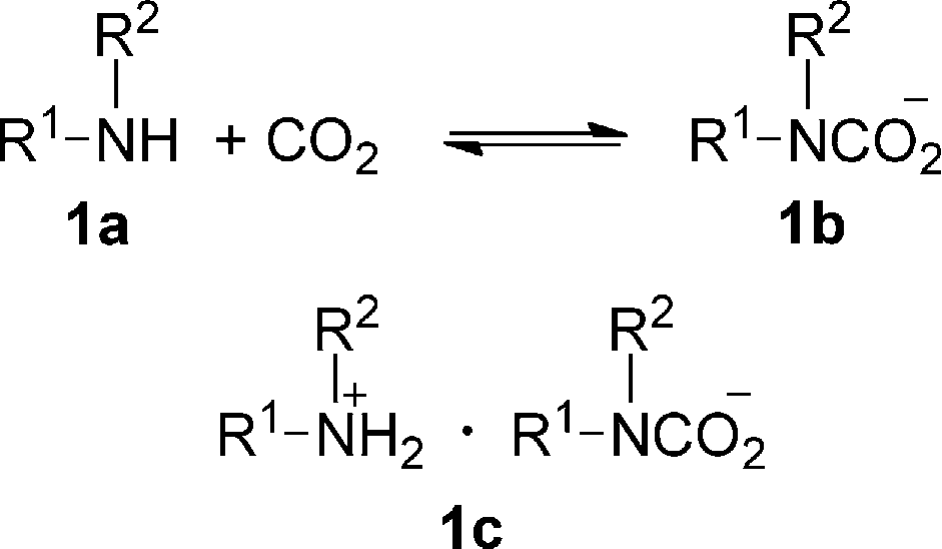
Formation of carbonated adducts (**1 b**) from amines (**1 a**) and corresponding ammonium carbamate self-derivative salts (**1 c**).

Amines (**1 a**) have a rare tendency to undergo aerial carbonation resulting in the formation of carbonated adducts (**1 b**). It is usually accepted[1a,b] that carbon dioxide has a low reactivity with amines forming unstable carbamic acids (**1 b**), which revert to their corresponding starting materials (Scheme 1).

Carbamic acid (or amino formic acid, NH_2_CO_2_H, R^1^=R^2^=H in **1 b**) is described as an unstable compound and, to the best of our knowledge, has not been synthesized or characterized by any experimental technique. Some structural aspects have been treated theoretically by quantum mechanical ab initio calculations.[2a,b] Carbamic acids are known only in the form of their salts, as ammonium carbamates (**1 c**), or their esters, referred to as carbamates or urethanes.

Ammonium carbamate (NH_4_NH_2_CO_2_, R^1^=R^2^=H in **1 c**) can directly be prepared by reaction between solid carbon dioxide (dry ice) and liquid ammonia. It is naturally found in the blood and urine of animals. Upon hydration, this white solid can lead to urea, a reaction first described by Bazarov in 1870 when ammonium carbamate was heated in a sealed glass tube at 130–140 °C.[3a,b] Therefore, ammonium carbamate is an intermediate in the manufacturing process of urea. It is also used as a nitrogen fertilizer.

Organic carbamates on the other hand are valuable synthetic intermediates[4a] and used in applications such as drug or agrochemicals synthesis. Like amides, they can form polymers such as polyurethane resins, and they are widely used as protecting groups of amines.[4b]

In the context of functional group protection, carbamic acids are considered unstable intermediates in solution that are formed during several standard deprotection protocols. Universally, free secondary and primary amines are obtained after standard work-up procedures, and the intermediate carbamic acids remain elusive.

Like the natural amino acid proline, prolinamide and some of its N-substituted derivatives have been used in enantioselective organocatalysis, often with greater efficiency than proline itself.[5a] Blackmond et al. have shown the importance of the solid state characteristics of proline itself with respect to its behavior as an organocatalyst.[5b] Surprisingly, not much is known about the solid-state structure of prolinamide. It is thus plausible that the formation of a stable carbamate self-derivative salt of prolinamide in the solid state should also affect its behavior as a catalyst.

**Figure 1:**
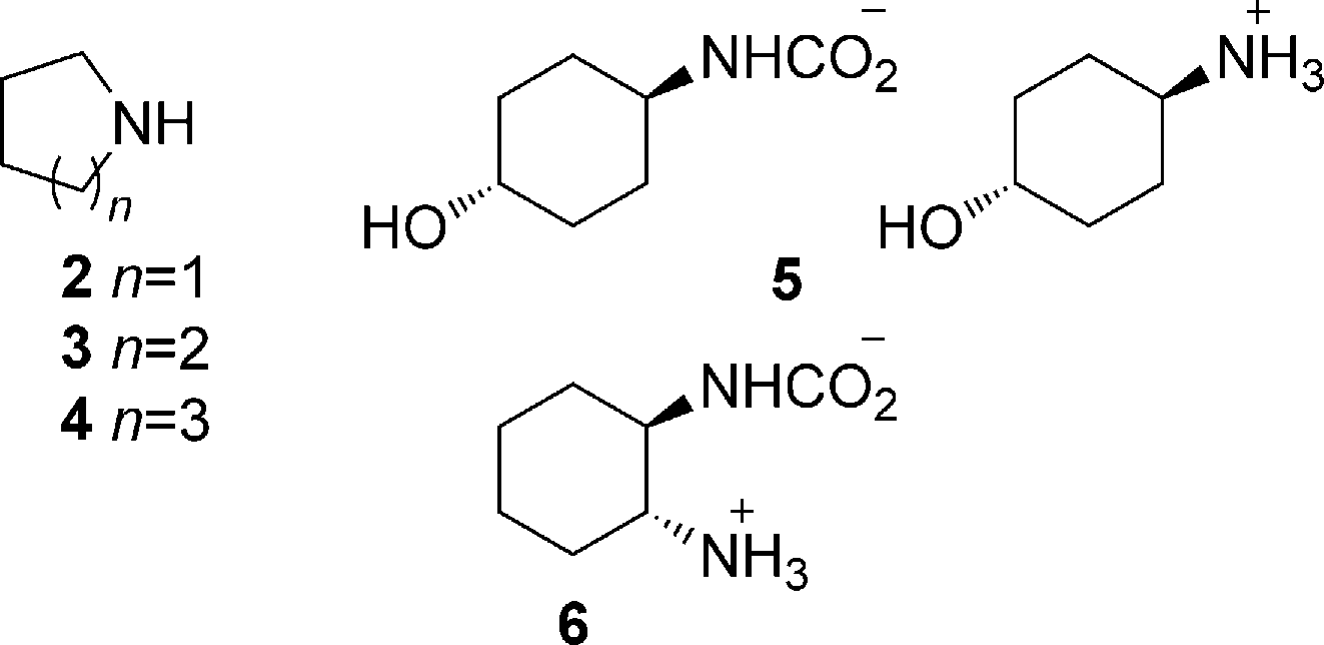
Selected ammonium carbamate self-derivatives reported in the literature.[6c, d]

Reactions of carbon dioxide with both primary (**1 a**, R^2^=H) and secondary amines have been reported and produce salts of the amine and the corresponding carbamic acid (**1 c**). Usually, powder or amorphous solids are formed, whereas diffraction-quality single crystals are more seldom. Single crystal structures of ammonium carbamate self-derivative salts of several cycloalkylamines have been reported (Figure 1), for example, pyrrolidine (**2**, CINZIJ[6a]), piperidine (**3**, COLQAW[6b]), azepane (**4**, COLQEA[6b]), 4-amino-cyclohexanol (**5**, ATUVEQ[6c]), and (1*R*,2*R*)-1,2-diaminocyclohexane (**6**, ZAVXOJ[6d]).

Hanessian et al.[6e] serendipitously observed that chiral diamine **6** slowly adsorbs CO_2_ and can be converted into crystalline carbamate salts, which exist as layered trihelicate structures. Diffracting crystals were obtained using “forced” carbonation. Similar carbamates have been obtained by Mondal[6b] from slow “natural” aerial carbonation of diamines in the presence of diols. Structures of benzylamine derivatives have also been obtained by Madarasz and co-workers[6f] from liquid amines in supercritical conditions, and have been identified and characterized by powder X-ray diffraction (PXRD), FTIR and HSQC ^1^H–^13^C direct coupled NMR.

As part of our interest for solid-state complexes and cocrystals,[6g] we are interested in further studies of prolinamide (ProNH_2_). Surprisingly, no crystal structure of this rather simple and common molecule has been published so far. The present work provides a possible explanation for this and yields, in addition to the structure of d-ProNH_2_, the crystal structures of two polymorphs of its carbonated adduct, the ammonium carbamate self-derivative salt of prolinamide, and a series of derived products, including dehydroprolinamide, a methylene-bridged prolinamide derivative, and a bicyclic derivative. This study emphasizes the reactivity of carbonated amines stabilized in the solid state and opens perspectives for a systematic study of reactions involving these trapped reactive species.

## Results and Discussion

**Figure 2:**
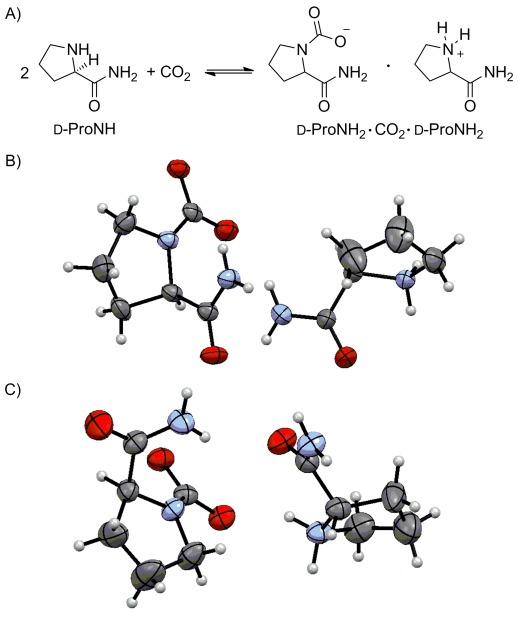
A) Reaction between two d-prolinamide and carbon dioxide leading to an ammonium carbamate self-derivative salt. Obtained single crystal structures of two polymorphs, B) form I and C) form II.

Serendipitously, we have been successful in obtaining single crystals of two polymorphs of a 1:1 molecular complex of the ammonium carbamate self-derivative salt of d-prolinamide (ProNH_2_–CO_2_^−^**⋅**ProNH_2_^+^, Figure 2). These forms were found by screening different crystals found in starting commercial samples. Carbonation of prolinamide influences the conformation of the molecules, as underlined by distinct N–C–C_(=O)_–N torsion angles (*T*1) in ProNH_2_^+^ and the ProNH_2_–CO_2_^−^ species (Table 1). In the carbonated ion, this torsion angle is close to 0°, bringing one of the hydrogen atoms of the exocyclic amide group relatively close to the endocyclic carbonated nitrogen atom (<2.40 Å).

**Table 1:** Values of the torsion angles (*T*1) for prolinamide (two molecules in the asymmetric unit) and the two polymorphs of its carbamate self-derivative salt

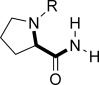	*T*1 [°]^[a]^	
	ProNH_2_^+^	ProNH_2_–CO_2_^−^
ProNH_2_–CO_2_^−^**⋅**ProNH_2_^+^ (Form I)	−147.3(2)^[b]^	3.0(3)^[b]^
ProNH_2_–CO_2_^−^**⋅**ProNH_2_^+^ (Form II)	−163.1(8)^[b]^	−0.4(12)^[b]^
ProNH_2_ (Molecule I)	−4.9(4)^[b]^	
ProNH_2_ (Molecule II)	−7.9(3)^[b]^	

[a] *T*1=N–C–C_(=O)_–N. [b] Standard uncertainties (s.u.) are given in parentheses.

**Scheme 2:**
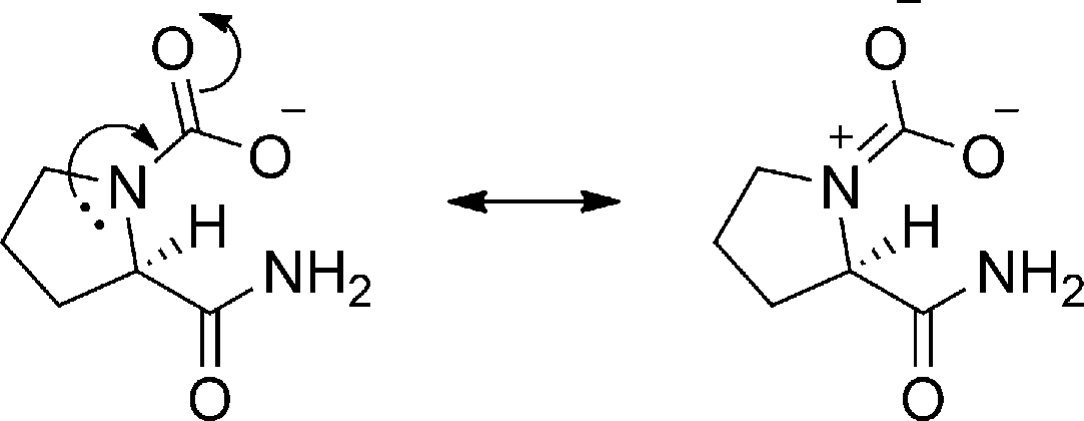
Resonance forms of the carbamate self-derivative of d-prolinamide.

The structure of the carbamate of prolinamide (ProNH_2_–CO_2_^−^) obtained by carbonation of prolinamide (ProNH_2_) is better described by resonance forms in which the lone pair of the nitrogen is delocalized on both oxygen atoms of the carbamate (Scheme 2). Indeed, in the crystal structures, both C–O bond lengths (≍1.25 Å) are equal and short, the C–N bond (≍1.38 Å) is shorter than a single C–N bond, the sum of valence angles around the nitrogen atom (≍119°+≍123°+≍124°) are close to 360°, and deviation of the nitrogen atom from the mean plane formed by surrounding atoms (carbon and oxygen atoms) is small and characteristic of sp^2^ hybridization.

**Figure 3:**
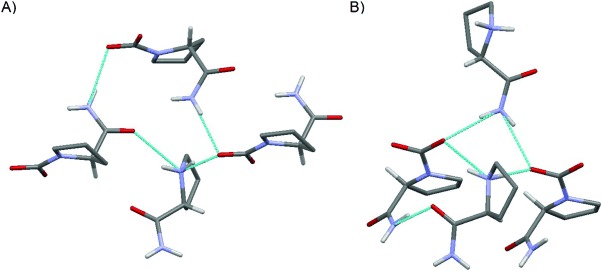
Hydrogen-bonding patterns (shown in cyan) within the two polymorphic structures A) form I and B) form II of the ammonium carbamate self-derivative salt of d-prolinamide (ProNH_2_–CO_2_^−^**⋅**ProNH_2_^+^).

The distinct conformations of the species in both polymorphic forms I and II (Table 1) allow for differences in crystal packing. In particular, hydrogen bonds (Table S1 in the Supporting Information) involve the two hydrogen atoms of the amide group, the two hydrogen atoms of the protonated ammonium nitrogen, one oxygen atom of the carbamate CO_2_ and, depending of the polymorph, either one oxygen atom of an extra carbamate (form II) or of another amide group (form I), in both cases leading to 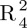
(8) clusters (Figure 3). These clusters of four entities, either three ProNH_2_–CO_2_ and one ProNH_2_^+^ (form I) or two ProNH_2_–CO_2_^−^ and two ProNH_2_^+^ (form II), are further linked by hydrogen bonds involving the remaining hydrogen-bond donor (NH_2_ of the amide) and acceptor (CO_2_ of the carbamate) sites to make extended sheets (Figure S1 in the Supporting Information).

**Figure 4:**
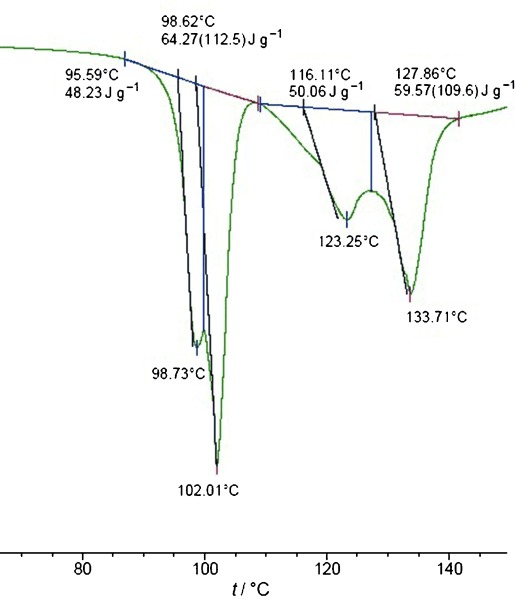
Calorimetric analysis (DSC) of commercial d-ProNH_2_ (temperature range: 30–160 °C). Four endotherms are visible in the first heating run.

Decarbonation of the carbamate salts (ProNH_2_–CO_2_^−^**⋅**ProNH_2_^+^) was monitored by calorimetry and confirmed stabilization of the reactive carbonated adduct in the solid state. Upon heating (30–160 °C) of samples of commercial d-ProNH_2_, four endotherms (fusion peaks) are observed in the first heating run (Figure 4 and S2 in the Supporting Information).

The high-temperature endotherms at 123.2 °C and 133.7 °C are attributed to the two polymorphic forms of the carbamate salts. The reported melting temperature of prolinamide being between 97–102 °C,[7] endotherms at 98.7 °C and 102.0 °C were attributed to d-ProNH_2_, suggesting presence in the starting material of a mixture of pure prolinamide and another derivative product or two polymorphs of prolinamide itself. Upon cooling, a single exotherm is observed at 71.5 °C, associated with recrystallization of the sample (Figure S2 a in the Supporting Information). The resulting powder melts at 98.2 °C (single endotherm, Figure S2 b).

The solid obtained after the first heating cycle corresponds to pure prolinamide, ProNH_2_, a result further confirmed by ^1^H and ^13^C NMR and crystallography (see the Supporting Information). This analysis hypothesizes that the starting commercial d-ProNH_2_ used has not been correctly conserved, and furnishes uncarbonated prolinamide and its carbonated product (stabilized as the ammonium carbamate salt). This study also indicates that upon melting (above 134 °C), decarbonation of the carbamate salt (ProNH_2_–CO_2_^−^**⋅**ProNH_2_^+^) occurs, leading to only prolinamide. Stabilization of the otherwise reactive carbamate in the solid state can thus be overcome by melting of the sample allowing escape of CO_2_ and recrystallization, upon cooling, of the free amine.

The same behavior was observed using different batches of d-ProNH_2_, from different commercial suppliers, suggesting that carbonation of this amine is a general, but overlooked process. This study also suggests that (at least) two polymorphs of prolinamide can be expected (close melting points at 98.7 and 102.0 °C); the most stable one melting at 98.2 °C.

Single crystals of d-ProNH_2_ were grown by sublimation of the commercially available material, which represents a mixture of prolinamide and its carbonated ammonium carbamate salt. X-ray diffraction analysis led to the first crystal structure of prolinamide itself, which can be considered as an otherwise common molecule (Figure S3 in the Supporting Information).

In the crystal structure, two molecules of d-ProNH_2_ are present in the asymmetric unit, adopting close but distinct conformations (Table 1). The amide group is extensively involved in stabilization of the crystal packing by forming hydrogen-bonded clusters (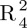
(8)) that form strands along a plane diagonal to crystallographic plane a–c. Hydrogen bonds observed in the two polymorphs of the ammonium carbamate self-derivative salt are quantitatively stronger than those observed in the crystal structure of d-ProNH_2_ alone (Table S2 in the Supporting Information), consistent with the measured melting points. Efforts to try to obtain another polymorphic form of d-ProNH_2_ failed so far.

The simulated powder X-ray diffraction (PXRD) data present characteristic peaks (Figure S4 in the Supporting Information): 2*θ*=8.0° (0,0,1) for d-ProNH_2_, 2*θ*=11.2° (1,0,1) for form I of the carbamate salt ProNH_2_–CO_2_^−^**⋅**ProNH_2_^+^, and 2*θ*=9.9° (0,0,2) for form II.

Experimental PXRD patterns collected at different temperatures for commercial samples of the prolinamide mixture with its carbonated product confirm our previous observations (Figure S5 in the Supporting Information). Before heating, diffraction peaks at 2*θ*=8° and 9.8° coexist. They are characteristic of a physical mixture of both d-ProNH_2_ and its ammonium carbamate salt (form II). Around 100 °C, the peak at 2*θ*=8° disappears (melting of d-ProNH_2_). The diffraction peak at 2*θ*=9.8° is present up to 130 °C and then disappears (complete melting of the sample). Upon cooling, the resulting powder only presents the peak characteristic of d-ProNH_2_ at 2*θ*=8°, confirming decarbonation of the carbamic acid.

**Figure 5:**
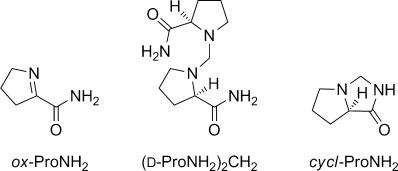
Compounds obtained from the ammonium self-derivative salt of prolinamide.

NMR analysis has also been employed to further characterize the samples and confirm the presence of the impurities contained within. After sublimation of commercial samples, ^1^H NMR of the obtained crystals clearly shows the presence of one major impurity (Figure S6 in the Supporting Information). We tentatively assigned the impurity as dehydroprolinamide (*ox*-ProNH_2_, Figure 5). ^1^H and ^13^C NMR data of the impurity are fully consistent with the dehydroprolinamide structure.[8] Quaternary carbon peaks were too weak in the product mixture to be unambiguously assigned. The proposed assignments are also consistent with ^1^H–^1^H COSY, DEPT-135 and ^1^H–^13^C HMQC experiments (data not shown).

**Figure 6:**
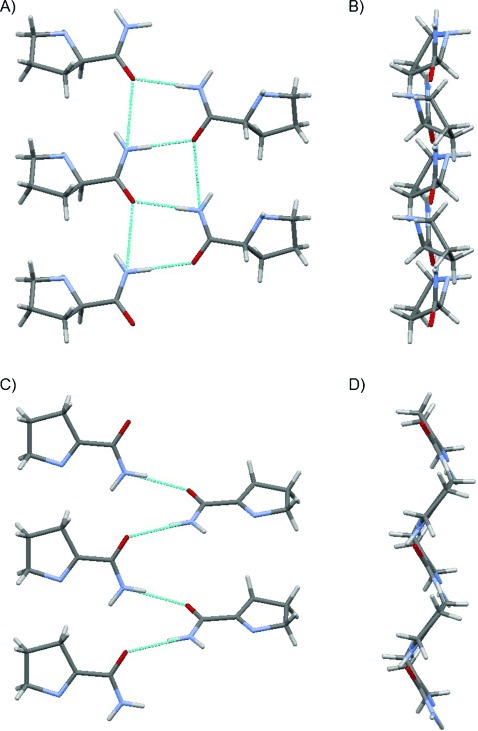
Hydrogen-bonding patterns (shown in cyan) stabilizing crystal structures of A) d-prolinamide and C) dehydroprolinamide (*ox*-ProNH_2_). Two perpendicular views are provided for each system (B and D, respectively).

In addition to dehydroprolinamide (*ox*-ProNH_2_), two other new solid products were found within recrystallized samples. Their structures have been determined by X-ray crystallography. We have thus obtained structures of *ox*-ProNH_2_, a methylene-bridged prolinamide ((ProNH_2_)_2_CH_2_), and a bicyclic derivative (*cycl*-ProNH_2_; Figure 5, Figure 6, Figure S7 in the Supporting Information). These derivatives can all potentially arise from reaction of the solid-state trapped ammonium carbamate self-derivative salt.

Hydrogen bonds in the crystal structures of ProNH_2_ and its oxidized product, *ox*-ProNH_2_, involve the amide group but are markedly distinct: while for ProNH_2_ hydrogen-bonded clusters (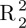
(8)) form strands (Figure 6 a), *ox*-ProNH_2_ adopts a similar arrangement but molecules are more tilted (Figure 6 b), the consequence being loss of a NH⋅⋅⋅O contact and a chain (C(4)) pattern of hydrogen bonds (Table S2 in the Supporting Information).

**Figure 7:**
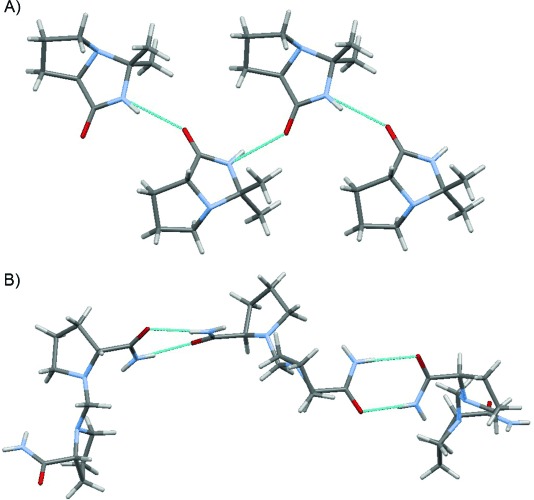
Hydrogen-bonding patterns (shown in cyan) stabilizing crystal structures of A) cyclic *cycl-*ProNH_2_ and B) bridged (ProNH_2_)_2_CH_2_) products.

**Figure 8:**
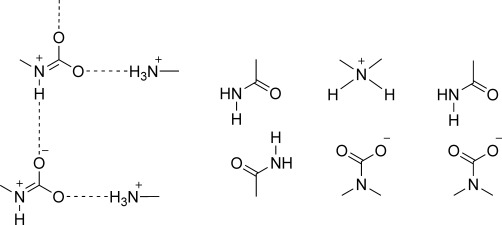
Hydrogen-bond interactions and synthon diversity in ammonium carbamate structures.

The presence of the carboxylic acid, amine and amide functional groups in the different structures also allows comparison of the hydrogen-bond geometry between these synthons. The cyclic hemi-aminal product, *cycl*-ProNH_2_, also forms chains (C(4)) that involve the amide functional group (Figure 7 A). The methylene-bridged product (ProNH_2_)_2_CH_2_), containing two amide groups, forms chains stabilized by homo-synthon (amide:amide) hydrogen bonds (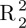
(8); Figure 7 B). Crystal structures of the ammonium carbamate (ProNH_2_–CO_2_^−^**⋅**ProNH_2_^+^) carbonated polymorphs reported here exhibit hydrogen-bonding patterns similar to those observed with other primary amide groups (Figure 8).[9a]^–c^

Interestingly, crystal structures of the different compounds obtained here display distinct amidic and/or non-amidic hydrogen bonding. This, in part, confirms the isosterism between ammonium carbamates and amides in terms of hydrogen bonds but also the competition between both functions for stabilizing the crystal packing. Indeed, our crystal structures containing ammonium carbamates, amides, and protonated (secondary) ammonium groups, cover different aspects of hydrogen bonding among those synthons.

## Conclusions

This study demonstrates that natural carbonation of amines is probably much more common than usually reported. The carbonation product, the ammonium carbamate self-derivative salt, can be stabilized in the solid state, as illustrated in the present work in the case of prolinamide (ProNH_2_). Indeed, combining differential scanning calorimetry (DSC), variable temperature powder X-ray diffraction (PXRD), NMR, and single crystallography, we unambiguously show that ProNH_2_ undergoes aerial carbonation upon normal storage, leading to a solid physical mixture of the free amine and its ammonium carbamate self-derivative salt. The complex behaves as a reversible solid-state CO_2_ sponge, opening potential applications if generalized to other amines.

The corresponding amine can be recovered intact after heating and recrystallization, a procedure necessary in our hands to obtain good quality crystals of prolinamide in order to solve its crystal structure.

The reactivity of carbonated amine, stabilized in the solid form, is also illustrated in the present work. Indeed, a series of new products were recovered that could originate from the reactive carbamic acid stabilized in the solid state, including dehydroprolinamide, a methylene-bridged prolinamide ((ProNH_2_)_2_CH_2_), and a bicyclic derivative. This opens interesting perspective for a systematic study of (solid-state) reactions involving trapped reactive carbonated amines.

The presence of carboxylic acid, amine and amide functions in the different structures further allowed comparison of the hydrogen-bond geometry and competition between these synthons. In particular, analysis of the crystal packings confirms isosterism of the ammonium carbamates and amides in terms of hydrogen bonds.

Finally, the importance of prolinamide and its derivatives in organocatalysis, and the known influence of solid-state properties on organocatalytic activity, led us to believe that these findings should also be considered when the catalytic activity of proline-derived amides is to be understood. Indeed, the ready carbonation and presence of considerable amounts of carbamate salts in the solid state (which does not occur for zwitterionic structures like proline) will have an effect on the catalytic activity. Furthermore, the possible presence of aminal derivatives such as *cycl*-ProNH_2_ is intriguing and clearly reminiscent of Seebach’s oxazolidinones, which are invariably present in proline-catalyzed reactions and have been shown to be competent intermediates in many organocatalytic reactions[10a] and exert a catalytic role by themselves.^[10b]^

## Experimental Section

**Materials**: d-Prolinamide (and l-prolinamide) was purchased from Sigma–Aldrich, TCI and Santa Cruz BioTechnologies. Solvents (essentially ethanol, 2-propanol, acetone) were purchased from Acros Organic (Geel, Belgium) and used with no further purification.

**X-ray crystallography**: Single crystal X-ray diffraction was performed on a Gemini Ultra R system (4-circle kappa platform, Ruby CCD detector) using Cu_Kα_ radiation *λ*=1.54056 Å. Selected crystals were mounted on the tip of a quartz pin using cyanoacrylate (commercial glue). Cell parameters were estimated from a pre-experiment run and full data sets collected at RT. Structures were solved by direct methods with sir92 (v.3.0)[11] program and then refined on F2 using SHELXL-97 software.[^12^] Non-hydrogen atoms were anisotropically refined and the hydrogen atoms (not implicated in hydrogen bonds) in the riding mode with isotropic temperature factors fixed at 1.2×*U*(eq) of the parent atoms (1.5× for methyl groups). Hydrogen atoms implicated in hydrogen bonds were localized by Fourier difference maps (ΔF). CCDC-927048 http://www.ccdc.cam.ac.uk/cgi-bin/catreq.cgi, 927050 http://www.ccdc.cam.ac.uk/cgi-bin/catreq.cgi(d-ProNH_2_**⋅**CO_2_^−^**⋅**d-ProNH_2_^+^ form I and II), 927064 http://www.ccdc.cam.ac.uk/cgi-bin/catreq.cgi(d-ProNH_2_), 927065 http://www.ccdc.cam.ac.uk/cgi-bin/catreq.cgi(*ox*-ProNH_2_), 927070 http://www.ccdc.cam.ac.uk/cgi-bin/catreq.cgi((d-ProNH_2_)_2_CH_2_) and (*cycl*-ProNH_2_) entries contain the supplementary crystallographic data for this paper. These data can be obtained free of charge from the Cambridge Crystallographic Data Centre via http://www.ccdc.cam.ac.uk/data_request/cif.

Ammonium carbamate self-derivative salts form I and II and methylene-bridged prolinamide single crystals have been recrystallized by slow evaporation in isopropanol, and bicyclic derivative single crystals in cyclohexane with MeOH. d-Prolinamide and dehydroprolinamide single crystals have been obtained after heating (sublimation) and recrystallization.

Crystal data for d-prolinamide: Monoclinic *P*2_1_ (n.4), *a*=10.7913(10) Å, *b*=5.2251(3) Å, *c*=11.9633(12) Å, *β*=113.085(11) °, *V*=620.54(11) Å^3^, *Z*=4, *D*_calc_=1.222 g cm^−3^, *µ(*Cu_K*α*_*)*=0.713 mm^−1^, *F(000)*=248, *θ_min_*=4.0° *to θ_max_*=67.4°, *R*=0.0527, *wR2*=0.1573, observed data *(I>*2*σI)*=1344, total=2542, *R*(int)=0.040, *S*=1.04.

**Powder X-ray diffraction (PXRD)**: Data were collected on a PANalytical reflexion-geometry diffractometer, using Ni-filtered Cu_Kα_ radiation *λ*=1.54179 Å at 40 kV and 40 mA with a X′Celerator detector. Each sample was analyzed between 4 and 50° 2*θ* with a step size of approximately 0.0167° 2*θ* and a total scan time of 3 min 48 s.

**Variable-temperature PXRD (VT-PXRD)**: Variable-temperature PXRD analysis was carried out using a D8 advance diffractometer (Bruker, Germany) (Cu_Kα_ *λ*=1.54178 Å, 40 kV, 30 mA) equipped with a Vantec (Bruker, Germany) detector.

**Differential scanning calorimetry (DSC)**: DSC analyses were carried out using a Q1000 DSC (TA Instruments, New Castle, USA) with a refrigerated cooling system (TA Instruments) and aluminum solid fat index (SFI) pans. Calibration was made with indium (mp: 156.6 °C) standard. Nitrogen was used as purge gas in order to prevent condensation in the cells. An empty aluminum SFI pan was used as reference. Samples weighed between 2 and 4 mg. Two cycles of heating were applied on the samples, between 30 and 160 °C at 5 °C min^−1^. The last cooling was at 20 °C min^−1^. All DSC analyses were carried out in duplicate. The integration and peak temperature measurements were performed using the Universal Analysis Software (version 4.2; TA Instruments). The melting peaks were integrated with a linear baseline.

**NMR spectroscopy**: ^1^H, ^13^C, ^1^H–^1^H COSY, DEPT-135 and ^1^H–^13^C HMQC spectra were obtained on a 400 MHz NMR (Jeol JNM EX-400) in CD_3_OH. Chemical shifts were reported in ppm according to tetramethylsilane using the solvent residual signal as an internal reference (CDCl_3_: H=7.26 ppm, C=77.16 ppm, DMSO-d_6_: H=2.50 ppm, C=39.52 ppm, D_2_O: H=4.79 ppm, Toluene-d_8_: H=2.09 ppm, C=20.40, THF-d_8_: H=3.58 ppm, C=67.57). Resonance multiplicity was described as m (multiplet). Carbon spectra were acquired with a complete decoupling for the proton.

## Supplementary material

As a service to our authors and readers, this journal provides supporting information supplied by the authors. Such materials are peer reviewed and may be re-organized for online delivery, but are not copy-edited or typeset. Technical support issues arising from supporting information (other than missing files) should be addressed to the authors.
