# Supplementary material for: Solid-State-Trapped Reactive Ammonium Carbamate Self-Derivative Salts of Prolinamide
Source: ChemistryOpen. 2013 Aug 12;2(5-6):194–9. doi: 10.1002/open.201300025 (PMC3892199; doi:10.1002/open.201300025)
Supplement: Supplementary file 1 [file open0002-0194-SD1.pdf]

ChemistryOPEN

Supporting Information

© 2013 The Authors. Published by Wiley-VCH Verlag GmbH & Co. KGaA, Weinheim

Solid-State-Trapped Reactive Ammonium Carbamate Self-Derivative Salts of Prolinamide

Anaëlle Tilborg, Steve Lanners, Bernadette Norberg, and Johan Wouters*[a]

open_201300025_sm_miscellaneous_information.pdf

Table S1. Selected geometry of H bonds for the two polymorphs of carbamate self-derivatives salt from prolinamide (D : donor, A : acceptor partner)

H-bonds	D···A distance (Å)	H···A distance (Å)	D-H···A angle (°)	Symmetry code
Form I				
N1 -- H1D ... O1	2.740(3)	2.30	110	
N1 -- H1D ... O2	2.948(3)	2.18	143	1/2+x, 5/2-y, 2-z
N1 -- H1E ... O4	2.618(2)	1.79	164	1/2+x, 3/2-y, 2-z
N2 -- H2D ... O3	2.797(3)	1.94	164	
N2 -- H2E ... O1	3.126(4)	2.42	144	-1/2+x, 5/2-y, 2-z
N4 -- H4A ... O4	2.839(3)	2.04	154	1-x, 1/2+y, 3/2-z
N4 -- H4B ... N3	2.731(3)	2.33	109	
N4 -- H4B ... O2	3.075(3)	2.32	146	1-x, -1/2+y, 3/2-z
Form II				
N1 -- H1D ... O3	2.704(10)	1.65	175	-1+x, y, z
N1 -- H1E ... O1	2.716(9)	2.15	107	
N2 -- H1E ... O4	2.903(9)	1.94	139	
N2 -- H2D ... O4	2.848(9)	1.93	158	1-x, -1/2+y, 1/2-z
N2 -- H2E ... O3	2.908(9)	2.18	163	2-x, -1/2+y, 1/2-z
N4 -- H4D ... O4	2.963(9)	1.98	161	2-x, -1/2+y, 1/2-z
N4 -- H4E ... O1	3.078(10)	2.08	152	

Table S2. Selected geometry of H bonds for Prolinamide (D : donor, A : acceptor partner)

H-bonds	D···A distance (Å)	H···A distance (Å)	D-H···A angle (°)	Symmetry code
N1 -- H1N ... O2	3.332(4)	2.47(5)	156(5)	2-x, 1/2+y, 1-z
N2 -- H2C ... O1	3.033(4)	2.46(5)	128(4)	x, 1+y, z
N2 -- H2C ... N1	2.730(5)	2.45(4)	101(4)	
N2 -- H2D ... O1	2.900(4)	1.92(3)	177.0(14)	1-x, 1/2+y, 1-z
N4 -- H4C ... O2	2.982(4)	2.00(4)	175(3)	2-x, 1/2+y, 1-z
N4 -- H4D ... O2	3.007(3)	2.36(4)	137(3)	x, 1+y, z
N4 -- H4D ... N3	2.738(4)	2.48(4)	100(3)	

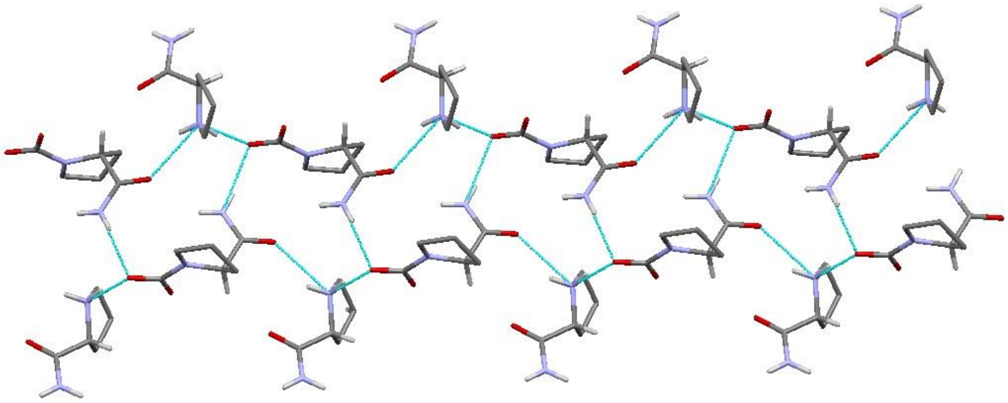

a)

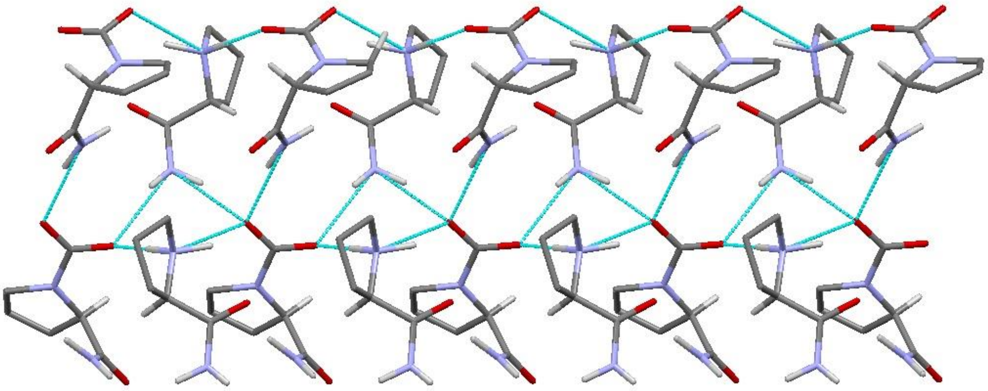

b)

Figure S1. H-bonding patterns within the two polymorphic structures of the ammonium carbamate self-derivative salt of D-prolinamide ($ProNH_2$-CO_2^- . $ProNH_2^+$). $R^2_4(8)$ clusters of four entities (either 3 $ProNH_2$-CO_2^- and 1 $ProNH_2^+$ (form I, a) or 2 $ProNH_2$-CO_2^- and 2 $ProNH_2^+$ (form II, b) are further linked by H-bond interactions involving the remaining H-bond donor (NH_2 of amide) and acceptor sites (CO_2 of carbamate) to make extended sheets.

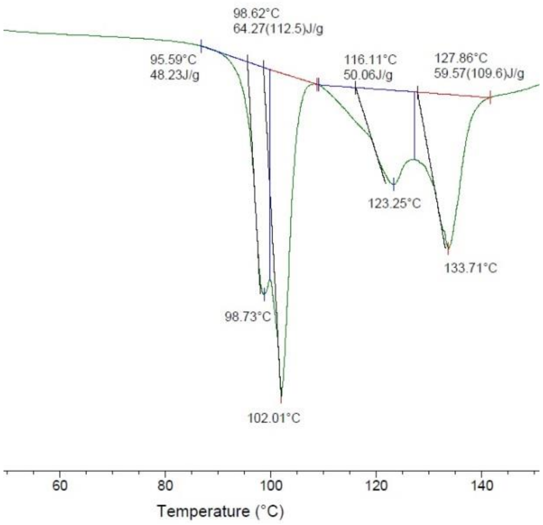

a)

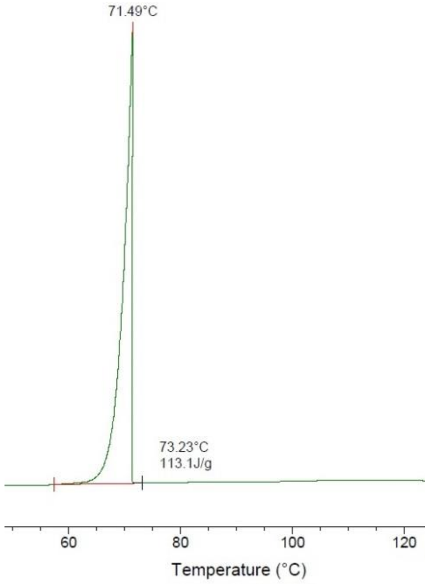

b)

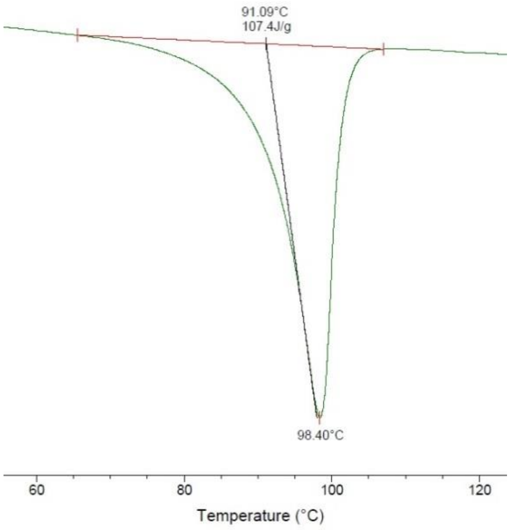

c)

Figure S2. Calorimetric analysis (DSC) of commercial D-ProNH_2 solid powders (30-160°C temperature range). Four endotherms are visible in the first heating run (a). Recrystallization exotherm in (b) and second heating run in (c).

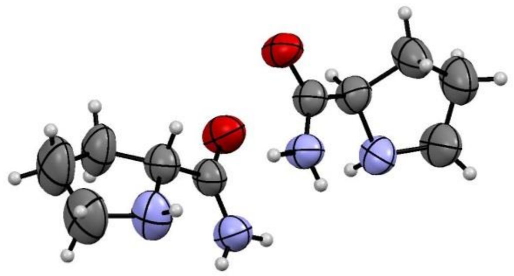

Figure S3. Single crystal structure of D-prolinamide (two molecules in the asymmetric unit).

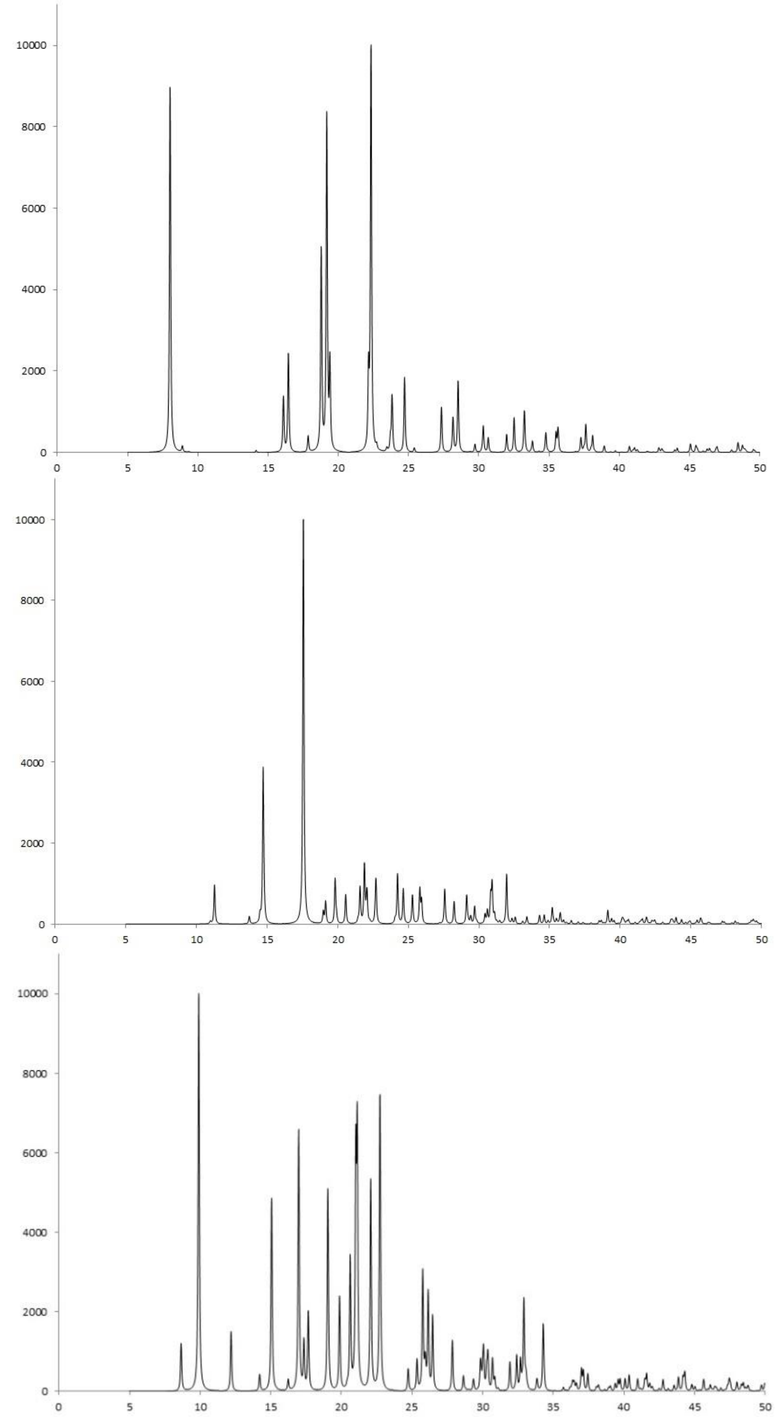

Figure S4. PXRD simulations for (from top to bottom) D-Prolinamide, D-ProNH$_2$-CO$_2^-$. D-ProNH$_2^+$ form I, D-ProNH$_2$-CO$_2^-$. D-ProNH$_2^+$ form II

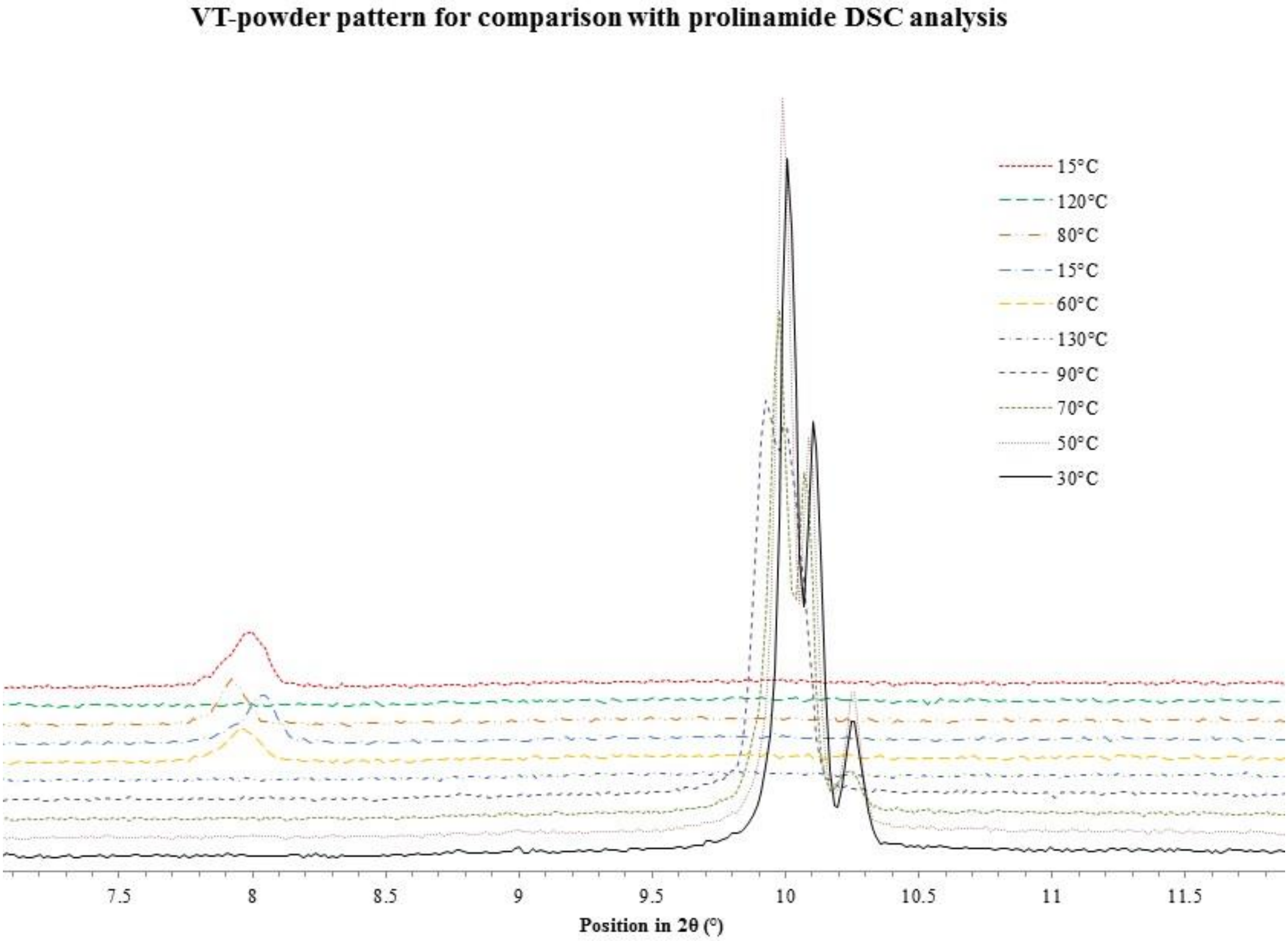

Figure S5. Powder X-ray diffraction (PXRD) diffractograms recorded at different temperature on a commercial sample of prolinamide, confirming the presence of a physical mixture of both D-ProNH$_2$ and its ammonium carbamate salt (form II). Before heating, diffraction peaks at 2θ = 8 and 9.8° are present, characteristic of both solids. Upon cooling of a heated sample, the resulting powder only presents the peak characteristic of D-ProNH$_2$ at 2θ = 8°, confirming decarbonation of the carbamic acid.

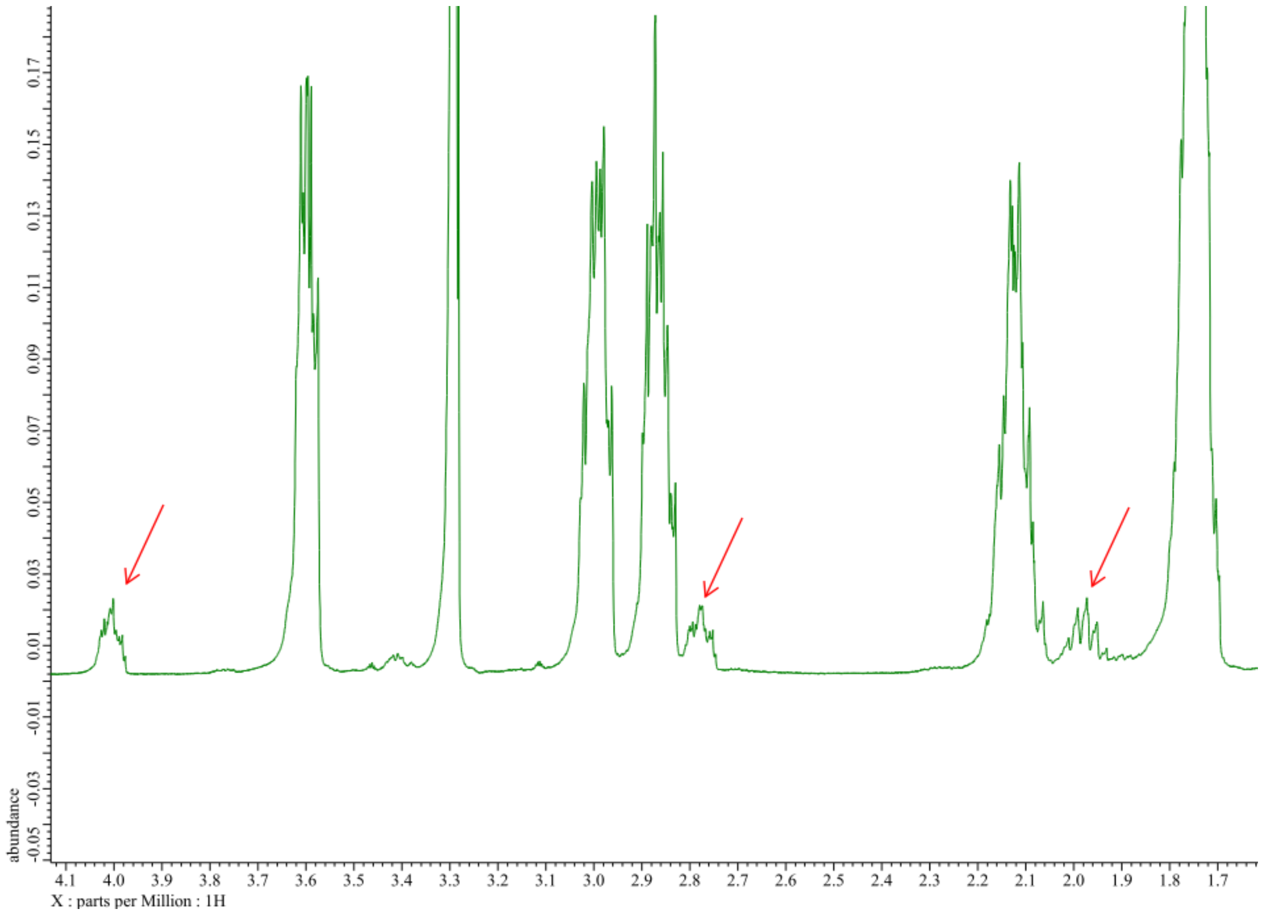

Figure S6. ^{1}H NMR analysis, and highlight of the occurrence after the sublimation of ox-ProNH$_2$, considered as an impurity in the prolinamide result powder mixture

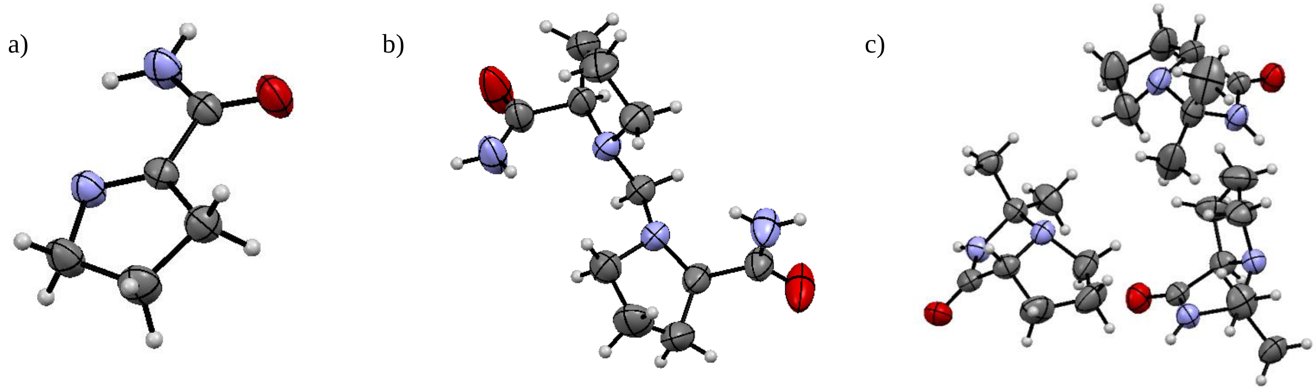

Figure S7. Single crystal structure of (a) dehydro-prolinamide (*ox*-ProNH$_2$), (b) methylene-bridged prolinamide (ProNH$_2$)$_2$CH$_2$), and (c) bicyclic derivative (*cycl*-ProNH$_2$) (three molecules in the asymmetric unit).
